# Institutional Needs

**Published:** 1918-01-19

**Authors:** 


### Institutional Needs.

### JARDOX, THE ALL-BRITISH FOOD.

(Jardox, Ltd., Crystal Palace Works, Anerley, London, S.E. 20.)

We have received a sample of this meat extract. A teaspoonful added to from 4 to 6 oz. of boiling water makes a refreshing and nutritive consommé. Jardox contains not only the extractive, stimulating constituents of meat, but also a certain proportion of its definite nutritive elements. Much literature has been written upon the vexed subject of the nutritive value of different so-called beef-teas; as a fact, in the light of recent work upon the nutritive effect of the amino acids and allied bodies, much of this requires reconsideration. The real test of the value of a meat extract is its clinical effect in low states of nutrition, and by this standard alone will the practising physician judge it. There can be no doubt that substances of the nature of the one before us give excellent clinical results, and are most valuable as dietetic agents in depressed states of nutrition. Jardox is pleasant to take, and has proved itself by many trials to be one of the best preparations of its kind. We think it is highly to be recommended, and has a very extended sphere of usefulness before it, both in the treatment of illness and also in domestic life under conditions of modern stress.

